# Redox Profile of Skeletal Muscles: Implications for Research Design and Interpretation

**DOI:** 10.3390/antiox12091738

**Published:** 2023-09-07

**Authors:** Olga Vasileiadou, George G. Nastos, Panagiotis N. Chatzinikolaou, Dimitrios Papoutsis, Dimitra I. Vrampa, Spyridon Methenitis, Nikos V. Margaritelis

**Affiliations:** 1 Department of Physical Education and Sports Science at Serres, Aristotle University of Thessaloniki, 62100 Serres, Greece; olgavasi@phed-sr.auth.gr (O.V.); nastosgg@phed-sr.auth.gr (G.G.N.); chatzinpn@phed-sr.auth.gr (P.N.C.); dpapoutsi@phed-sr.auth.gr (D.P.); 2 Department of Nutrition Sciences and Dietetics, Faculty of Health Sciences, International Hellenic University, 57001 Thessaloniki, Greece; dimitravraba@gmail.com; 3 School of Physical Education and Sports Science, National and Kapodistrian University of Athens, 15772 Athens, Greece; smetheni@phed.uoa.gr

**Keywords:** antioxidants, enzymes, fibers, oxidative stress, redox, skeletal muscle

## Abstract

Mammalian skeletal muscles contain varying proportions of Type I and II fibers, which feature different structural, metabolic and functional properties. According to these properties, skeletal muscles are labeled as ‘red’ or ‘white’, ‘oxidative’ or ‘glycolytic’, ‘slow-twitch’ or ‘fast-twitch’, respectively. Redox processes (i.e., redox signaling and oxidative stress) are increasingly recognized as a fundamental part of skeletal muscle metabolism at rest, during and after exercise. The aim of the present review was to investigate the potential redox differences between slow- (composed mainly of Type I fibers) and fast-twitch (composed mainly of Type IIa and IIb fibers) muscles at rest and after a training protocol. Slow-twitch muscles were almost exclusively represented in the literature by the soleus muscle, whereas a wide variety of fast-twitch muscles were used. Based on our analysis, we argue that slow-twitch muscles exhibit higher antioxidant enzyme activity compared to fast-twitch muscles in both pre- and post-exercise training. This is also the case between heads or regions of fast-twitch muscles that belong to different subcategories, namely Type IIa (oxidative) versus Type IIb (glycolytic), in favor of the former. No safe conclusion could be drawn regarding the mRNA levels of antioxidant enzymes either pre- or post-training. Moreover, slow-twitch skeletal muscles presented higher glutathione and thiol content as well as higher lipid peroxidation levels compared to fast-twitch. Finally, mitochondrial hydrogen peroxide production was higher in fast-twitch muscles compared to slow-twitch muscles at rest. This redox heterogeneity between different muscle types may have ramifications in the analysis of muscle function and health and should be taken into account when designing exercise studies using specific muscle groups (e.g., on an isokinetic dynamometer) or isolated muscle fibers (e.g., electrical stimulation) and may deliver a plausible explanation for the conflicting results about the ergogenic potential of antioxidant supplements.

## 1. Introduction

Mammalian skeletal muscles are quite heterogeneous, containing muscle fibers with distinct structural (e.g., myosin heavy chain isoform, motor unit and fiber size, capillary and mitochondrial density), metabolic (e.g., myoglobin content, ATP production source, oxidative capacity) and functional (e.g., contraction velocity, force production, rate of fatigue development) properties [1]. Due to these characteristics, skeletal muscles are frequently labeled as ‘red’ or ‘white’, ‘oxidative’ or ‘glycolytic’, ‘slow-twitch’ or ‘fast-twitch’ depending on the ratio of the different fiber types they contain. Two major advantages that result from this heterogeneity between muscle fibers are, first, the ability of a skeletal muscle (containing both muscle fiber types) to participate in activities ranging from low-intensity efforts to fast and maximal contractions and, second, to demonstrate remarkable adaptability in response to diverse external (e.g., exercise training, environmental changes) and internal (e.g., substrate availability, inflammation) stimuli [2,3].

Few data exist in the literature regarding the redox profile of muscle fibers (e.g., Type I compared to Type II) or whole muscles (e.g., soleus compared to extensor digitorum longus) predominantly from studies using animal models (e.g., rodents) under diverse conditions, such as aging and disease [4,5]. Regarding exercise, which is probably the most potent physiological and non-pharmacological stimulus challenging redox homeostasis, the relevant data is fragmented [6,7]. This is of major importance, bearing in mind that redox processes are increasingly recognized as a fundamental part of skeletal muscle metabolism at rest as well as during and after exercise [8,9,10,11]. Thus, the purpose of the present review is to gather and synthesize, for the first time, the relevant data, and more specifically, the aim is three-fold: (i) to review the available literature about the redox properties of slow-twitch (oxidative) and fast-twitch (glycolytic) skeletal muscles at rest; (ii) to examine the respective redox profiles after chronic exercise training; and (iii) to present potential implications for future research design and interpretation.

Towards these aims and taking into consideration the wide methodological heterogeneity among exercise redox biology studies, the data presented herein were included irrespective of the analytical method applied for the assessment of redox status (e.g., ELISA, spectrophotometry or HPLC), the exercise training protocol implemented (e.g., resistance or endurance, continuous or interval) or the animal model used (mainly rats) in the original studies. Moreover, given the exploratory nature of the present review and the fact that most of the original studies did not investigate the redox differences between slow- and fast-twitch muscles but rather examined the differences between young and old, fed and unfed or trained and untrained groups, we focused on synthesizing the available descriptive data (i.e., percent differences) and did not perform further analyses. Finally, since most original studies presented their data only in figures (i.e., bar or line graphs), we used the WebPlotDigitizer web-based tool to extract the relevant data in spreadsheets [12].

## 2. Redox Profile of Skeletal Muscles

### 2.1. Catalase

Catalase is regarded as the ‘oldest’ antioxidant enzyme and catalyzes the decomposition of hydrogen peroxide into water and oxygen [13]. Taking into account that hydrogen peroxide features signaling properties regulating some of the most commonly investigated exercise adaptations, catalase received great attention in exercise redox biology studies. Most of the studies in the literature measured the activity of catalase, while only a few studies assessed gene expression via mRNA levels. Regarding enzyme activity, muscle oxidative capacity and catalase seem to be strongly associated since, in the majority of studies, slow-twitch muscles (i.e., muscles with the most Type I fibers and higher oxidative capacity) exhibited higher levels of catalase activity compared to fast-twitch muscles. In particular, the most characteristic and widely used oxidative muscle, the soleus, which features approximately 70–80% Type I fibers, demonstrated 300–745% higher catalase activity compared to the glycolytic region of the fast-twitch gastrocnemius muscle and 60–210% higher activity compared to its oxidative part [14,15,16,17,18], 25–410% higher activity compared to the extensor digitorum longus [6,7,14,19,20], 110–420% and 630% higher activity compared to the deep vastus lateralis and the superficial vastus lateralis, respectively [21,22], and finally 175% higher activity compared to the epitrochlearis [7]. It should be clarified that all the aforementioned muscles (i.e., glycolytic and oxidative gastrocnemius, extensor digitorum longus, deep and superficial vastus lateralis and epitrochlearis) are categorized as fast-twitch (Type IIa or IIb) and are, therefore, compared throughout the paper with the slow-twitch soleus muscle. Regarding mRNA levels of catalase, the results are conflicting, showing either similar or higher (up to 170%) levels in the glycolytic and oxidative gastrocnemius compared to the soleus, while the latter exhibited 300% higher catalase mRNA levels compared to the extensor digitorum longus [16,23,24].

### 2.2. Superoxide Dismutase

The discovery of the unique enzymatic activity of superoxide dismutase, namely the catalysis of the dismutation of superoxide radical into molecular oxygen and hydrogen peroxide, has been one of the milestones in redox biology history [25]. As the first enzymatic line of defense controlling the metabolism of the parent reactive oxygen species (i.e., superoxide), a large amount of work has been performed on superoxide dismutase, which has been assessed in exercise studies either as total SOD or separately for the two intracellular isoforms (i.e., CuZn-SOD and Mn-SOD). SOD activity in the soleus was higher compared to the glycolytic (40–425%) and oxidative gastrocnemius (10–195%) [15,16,18,24,26] as well as higher compared to the rectus femoris (60%) [27], deep vastus lateralis (from minor difference up to 115%) [21,22,28], superficial vastus lateralis (100%), plantaris (80%) [22] and epitrochlearis (40%), while it was similar to the extensor digitorum longus [7]. It should, however, be mentioned that occasionally the mixed and oxidative gastrocnemius have been reported to exhibit slightly higher (20% and 10%, respectively) SOD activity compared to the soleus [16,27]. Within the same muscle, differences have also been reported between heads or regions with different oxidative characteristics. For instance, the oxidative gastrocnemius exhibited 30–120% higher SOD activity compared to the glycolytic gastrocnemius [15,16,18,24,26], the medial head of triceps brachii has 70% and 35% greater SOD activity compared to the glycolytic and oxidative long heads, respectively [15], while superficial vastus lateralis exhibited slightly higher SOD activity compared to deep vastus lateralis [22].

Regarding specific SOD isoforms, CuZn-SOD activity was 235% higher in the soleus compared to the glycolytic gastrocnemius [17], 15–150% higher compared to the deep vastus lateralis and 125% higher compared to the superficial vastus lateralis [22,28], 65% higher compared to the extensor digitorum longus [19] and 95% higher compared to the plantaris [22]. Likewise, CuZn-SOD mRNA levels in the soleus are up to 130% higher compared to the glycolytic gastrocnemius [16,24], 200% higher compared to the deep vastus lateralis and almost similar compared to the superficial vastus lateralis [22], 35% higher compared to the extensor digitorum longus [23] and 40% higher compared to the plantaris [22]. Interestingly, CuZn-SOD mRNA levels are equal to or up to 40% higher in the oxidative gastrocnemius compared to the soleus and 40–140% compared to the glycolytic gastrocnemius [16,24].

Regarding Mn-SOD activity, the soleus exhibited 150% higher activity compared to the glycolytic gastrocnemius [17], 30% and 45% higher compared to the superficial vastus lateralis and the plantaris, respectively [22], and 20% higher compared to the extensor digitorum longus [19]. The results are conflicting for the deep vastus lateralis, which exhibits both higher by 42% [28] and lower by 20% Mn-SOD activity compared to the soleus [22]. Mn-SOD mRNA levels are 67% higher in the soleus compared to the deep vastus lateralis [22], lower by 155% and 110–195% compared to the superficial vastus lateralis [22] and oxidative gastrocnemius [16,24], respectively, and similar to the extensor digitorum longus [23]. The soleus exhibits similar [29] or higher Mn-SOD mRNA levels [22] compared to the plantaris, whereas there are contradictory findings concerning the glycolytic gastrocnemius, as it presents either 185% higher [24] or 55% lower Mn-SOD mRNA levels compared to the soleus [16].

### 2.3. Peroxiredoxins

Peroxiredoxins (Prx) are a family of antioxidant enzymes that control peroxide levels and fine-tune signal (redox) transduction in mammalian cells [30,31], while they have also been implicated in exercise metabolism [32,33]. We found only two studies to date that have assessed the mRNA levels of several peroxiredoxin isoforms in slow-(soleus) and fast-twitch (extensor digitorum longus and epitrochlearis) muscles [7,23]. In the first study, mRNA levels of Prx3, Prx5 and Prx6 were almost similar between the soleus and the extensor digitorum longus under control conditions [23]. In the second study, no conclusion can be drawn given that the data in the original manuscript are presented only for the experimental group (i.e., a high-fat diet-treated group of rats) in relation (fold change) to the control condition (i.e., standard diet) [7]. Thus, we could not calculate the difference between the three muscles (i.e., soleus, extensor digitorum longus and epitrochlearis) in terms of peroxiredoxin mRNA levels under control conditions. Given the key role of peroxiredoxins in hydrogen peroxide sensing and signal transduction [31], further studies are warranted to investigate the potential differences in the activity, content and mRNA levels of this family of enzymes among different muscles and in relation to exercise.

### 2.4. Glutathione and Related Enzymes

Glutathione is the most abundant non-protein thiol in the erythrocyte (≈1.7 mM; [34]) and serves diverse direct and indirect antioxidant roles [35]. In particular, it acts as a direct scavenger, as a substrate for glutathione peroxidase and as a recycling agent of vitamin C, which is interdependent with vitamin E. Thus, its role in exercise redox metabolism is central.

Herein, we will first present the available information on the reduced (GSH) and oxidized (GSSG) forms of glutathione as well as on their ratio and then will review the relevant data on its closely associated enzymes, namely glutathione reductase and peroxidase. Glutathione in the soleus was found to be up to 215% higher than in the deep vastus lateralis [21,28], 105% higher than the extensor digitorum longus and 18% higher than the epitrochlearis [7]. Some studies measured total glutathione and not the reduced or oxidized form separately and reported that total GSH in the extensor carpi radialis was slightly greater compared to the oxidative gastrocnemius, triceps and splenius, while the oxidative gastrocnemius exhibited 15% and 195% higher levels compared to the mixed vastus lateralis and longissimus dorsi, respectively [36]. Furthermore, the soleus exhibited from minor up to 200% greater total GSH concentration compared to the deep vastus lateralis [21,28]. Regarding glutathione disulfide, the oxidized form of glutathione (GSSG), the two aforementioned studies reported conflicting results, with the first presenting 25–50% higher values in the deep vastus lateralis than the soleus and the latter featuring 100% greater concentration in the soleus compared to the deep vastus lateralis [21,28]. Nevertheless, the soleus has a 50–110% higher GSH to GSSG ratio than the deep vastus lateralis [21,28]. Regarding reduced thiols, the soleus exhibited 37% to 134% and 20% to 42% higher concentrations compared to the glycolytic and oxidative gastrocnemius, respectively [16,24].

The activity of glutathione reductase (GR), the enzyme responsible for the recycling of GSH from GSSG using NADPH as substrate [37], in the oxidative gastrocnemius was 15%, 55%, 114%, 65% and 45% higher compared to the extensor carpi radialis, triceps, splenius, mixed vastus lateralis and longissimus dorsi, respectively [36], while the soleus exhibited 70–115% higher GR activity than the deep vastus lateralis [21,28]. The activity of glutathione peroxidase (GPx), which is critically involved among other functions in the reduction of H_2_O_2_ and lipid hydroperoxides [38], was found to be higher in the soleus compared to a large number of fast-twitch muscles, such as the glycolytic gastrocnemius (30–2680%), the oxidative gastrocnemius (5–290%), the mixed gastrocnemius (400%), the rectus femoris (2190%), the deep (180–690%) and superficial vastus lateralis (1765%) and the extensor digitorum longus (255%), as reported in several studies [15,16,17,18,19,21,22,24,26,27,28,39]. The oxidative gastrocnemius, the more oxidative head of the gastrocnemius, exhibited higher GPx activity than the extensor carpi radialis (42%), triceps (55%), MVL (118%), splenius (55%), longissimus dorsi (19%) and glycolytic gastrocnemius (74–1215%) [15,16,18,24,26,36]. Finally, the medial head of triceps brachii has 485% and 90% higher GPx activity compared to the glycolytic and oxidative long heads, respectively [15]. Regarding GPx mRNA levels, the oxidative gastrocnemius, glycolytic gastrocnemius and soleus presented almost the same mRNA levels [24]. However, GPx1 mRNA levels in the soleus were found to be 30% higher compared to the oxidative gastrocnemius, 1120% compared to the extensor digitorum longus, and up to 1310% compared to the glycolytic gastrocnemius [16,23]. As for GPx3 mRNA levels, the soleus exhibited 40% and 130% higher levels compared to the oxidative and glycolytic gastrocnemius, respectively [16]. Finally, thioredoxin reductase activity, which is dependent upon NADPH levels, in the soleus was 160% and 400% higher compared to the extensor digitorum longus and epitrochlearis, respectively [7].

### 2.5. Oxidation Products

Oxidation products are the most commonly used biomarkers (also known as ‘fingerprints’) of oxidative stress used in the literature and are actually the products of the reaction between reactive species and biomolecules, such as proteins, lipids and DNA [40]. Despite their wide use, oxidative stress biomarkers have been criticized for their limited mechanistic insights (e.g., which reactive species has been involved, which pathway has been affected). However, their potential to report on redox status and monitor a condition longitudinally serves as a major advantage. Two oxidative stress biomarkers, a lipid peroxidation product (malondialdehyde; MDA) and a protein oxidation product (protein carbonyls), have been measured in slow- and fast-twitch muscles. In all studies, MDA was assessed via thiobarbituric acid reactive substances (TBARS), which have been highly criticized as biomarkers of oxidative stress [41]; however, these analytical issues are beyond the scope of the present review. Based on the available literature, MDA levels in the soleus were 2385% higher compared to the mixed gastrocnemius [42], 20–570% higher than the glycolytic gastrocnemius [17,26], 15% higher than the oxidative gastrocnemius [26], 10–415% higher than the deep vastus lateralis [21,28] and 68% higher than the vastus lateralis [42]. Based on these data, it could be argued that slow-twitch muscles seem to present higher levels of MDA; however, some studies reported 55% and 60% higher levels in the plantaris muscle [29] and in the extensor digitorum longus [42], which are both fast-twitch muscles, compared to the soleus, respectively. Regarding protein carbonyls, to the best of our knowledge, only one study measured their concentration in rat mitochondria from the tibialis anterior muscle (which is mainly composed of fast-twitch muscle fibers) and was found to be 22% higher compared to the respective values in the soleus [43].

### 2.6. Reactive Species Production

Identifying the cellular sources and the precise reactive species produced under diverse conditions, as well as quantifying their concentration, are of paramount importance in defining their role in biology [44,45]. NADPH oxidases and mitochondria are considered the major sources of reactive species in skeletal muscles and other tissues [46,47,48]. Regarding the activity of NADPH oxidases as assessed by hydrogen peroxide (H_2_O_2_) production, the soleus exhibited minor up to 60% higher activity compared to the oxidative gastrocnemius, whereas the findings for the soleus relative to the glycolytic gastrocnemius are controversial, ranging from −95% to +140% activity [16,24]. With respect to mRNA levels of NADPH oxidases in these muscles, data are again controversial between studies, reporting either 115% and 65% higher or 15% and 60% lower NOX2 mRNA levels in the soleus compared to the glycolytic and oxidative gastrocnemius, respectively [16,24]. Regarding NOX4 mRNA levels, the soleus exhibited similar or 170% higher levels compared to the glycolytic gastrocnemius, whereas the respective levels were either 60% higher or 40% lower compared to the oxidative gastrocnemius. Finally, dual oxidase 1 (an oxidase that produces hydrogen peroxide; DUOX1) mRNA levels were 20% higher and almost similar in the soleus compared to the glycolytic and oxidative gastrocnemius, respectively [16]. Similar to peroxiredoxins, data could not be extracted from Pinho et al. (2017) [7] concerning the expression of the different subunits of the NADPH oxidases (i.e., gp91phox, p47phox and p67phox). Beyond NADPH oxidases, three different studies measured H_2_O_2_ production/emission from mitochondria in slow- and fast-twitch muscles [6,7,43]. More specifically, the tibialis anterior demonstrated 166%, the extensor digitorum longus 116% (normalized for citrate synthase 400%), the epitrochlearis 185% and the mixed gastrocnemius 90% greater mitochondrial H_2_O_2_ production compared to the soleus. Along with the soleus, Picard et al. (2012) [6] also used the adductor longus, another slow-twitch muscle. The extensor digitorum longus and the mixed gastrocnemius exhibited 130% and 105% greater mitochondrial H_2_O_2_ production compared to the adductor longus, respectively. No difference was found between the two slow-twitch muscles, the soleus and the adductor longus. Finally, the study by Oyenihi et al. (2019) [42] quantified total reactive species production via a 2′,7′-dichlorodihydrofluorescein (DCF) probe and reported 35% and 145% higher production in the soleus compared to the extensor digitorum longus and gastrocnemius, and similar production between the soleus and the vastus lateralis. Apparently, further (exercise) studies are needed in an effort to reveal the exact reactive species that are involved in specific skeletal muscle responses and adaptations. However, this requires sophisticated and expensive tools (i.e., biosensors and techniques) and specialized analytical skills.

### 2.7. Brief Synopsis

Based on the available literature (Table 1), it seems that at rest (i) antioxidant enzymes exhibit higher activity in slow-twitch (oxidative) skeletal muscles compared to fast-twitch (glycolytic) muscles; (ii) even within a skeletal muscle, heads or regions with higher oxidative capacity (e.g., oxidative vs. glycolytic gastrocnemius, deep vs. superficial vastus lateralis or triceps brachii oxidative vs. glycolytic long head) present higher antioxidant enzyme activity; (iii) the data about the mRNA levels of redox enzymes (e.g., SOD, GPx and NADPH oxidases) are conflicting between slow- and fast-twitch skeletal muscles, even for the different isoforms, such as Mn-SOD versus CuZn-SOD and NOX2 versus NOX4; (iv) slow-twitch muscles exhibit higher content of glutathione and reduced thiols compared to fast-twitch muscles; (v) slow-twitch muscles exhibit higher levels of lipid peroxidation (as assessed by the questionable TBA assay); (vi) fast-twitch muscles exhibit higher mitochondrial H_2_O_2_ production compared to slow-twitch muscles.

## 3. Redox Profile of Skeletal Muscles after a Training Intervention

### 3.1. Catalase

As with untrained conditions, muscles with higher oxidative capacities presented higher catalase activity levels compared to less oxidative/glycolytic muscles. More specifically, the soleus predominates as the muscle with the highest catalase activity in comparison with the glycolytic gastrocnemius (90–530%) [15,17,24], the oxidative gastrocnemius (145%) [15], the deep vastus lateralis (99–125%) [21,22], the superficial vastus lateralis (335%) [22] and the extensor digitorum longus (170%) [20]. Interestingly, even within the same muscle, heads with different oxidative properties exhibit different redox characteristics. In particular, the oxidative gastrocnemius has 155% higher catalase activity compared to the glycolytic gastrocnemius, and the triceps brachii oxidative long head has 100% higher activity compared to the glycolytic long head, while the triceps brachii medial head has 115% and 335% higher catalase activity compared to oxidative and glycolytic long heads, respectively [15]. Regarding catalase mRNA levels after the training interventions, data are scarce showing that the glycolytic gastrocnemius and the soleus exhibit almost identical levels; however, the oxidative gastrocnemius exhibits more than 120% higher mRNA levels compared to the glycolytic gastrocnemius and the soleus [24].

### 3.2. Superoxide Dismutase

After exercise training, the soleus exhibited 80–290% higher SOD activity compared to the glycolytic gastrocnemius and up to 100% higher compared to the oxidative gastrocnemius [15,24,26], 45–65% compared to the mixed gastrocnemius, 100–110% more than the rectus femoris [27] as well as 160% and 130% more than the plantaris and the superficial vastus lateralis, respectively [22]. Data are conflicting regarding SOD activity between the soleus and the deep vastus lateralis, with some studies reporting 18–50% higher levels in the soleus and other studies showing 25–38% higher levels in the deep vastus lateralis [21,22,28]. Of note, SOD activity in the oxidative gastrocnemius was 40–290% higher compared to the glycolytic gastrocnemius [15,24,26], while the triceps brachii medial head presented 195% and 90% higher SOD activity compared to the glycolytic and oxidative long head, respectively [15].

Regarding the different SOD isoforms, exercise training also induced significant increases in both CuZn-SOD activity and CuZn-SOD mRNA levels. The post-training soleus exhibited 400% higher CuZn-SOD activity compared to the glycolytic gastrocnemius [17], 185% higher values compared to the plantaris and 125% compared to the superficial vastus lateralis [22]. Also, in some studies, the deep vastus lateralis exhibited 25% higher CuZn-SOD activity compared to the soleus, while in others, it was 75% lower [22,28]. With regard to CuZn-SOD mRNA levels, the oxidative gastrocnemius showed 1720% and 100% greater values compared to the glycolytic gastrocnemius and the soleus, respectively, whereas soleus exhibited 810% greater compared to the glycolytic gastrocnemius [24]. Finally, the soleus exhibited 215%, 145% and 40% greater CuZn-SOD mRNA levels against the deep vastus lateralis, the plantaris and the superficial vastus lateralis, respectively [22].

Likewise, post-training Mn-SOD activity was 15–25% higher in the deep vastus lateralis against the soleus [22,28], as well as 540% and 75% higher in the soleus compared to the glycolytic gastrocnemius [17] and the superficial vastus lateralis [22], respectively. Similar to CuZn-SOD, post-training Mn-SOD mRNA levels in the oxidative gastrocnemius were higher by 45% and 98% compared to the soleus and the glycolytic gastrocnemius, respectively, and 35% higher in the soleus compared to the glycolytic gastrocnemius [24]. The soleus also exhibited 35% and 90% higher Mn-SOD m-RNA levels compared to the deep vastus lateralis and the plantaris, respectively [22].

### 3.3. Glutathione and Related Enzymes

After the training period, conflicting results have been reported both for the reduced and total GSH levels between the soleus and the deep vastus lateralis [21,28]. Regarding total GSH, 38%, 40% and 55% higher levels were found in the oxidative gastrocnemius compared to the extensor carpi radialis, triceps and splenius, respectively, 20% greater than the mixed vastus lateralis and 205% than the longissimus dorsi [36]. Yet, the mixed vastus lateralis had 160% higher total GSH values than the longissimus dorsi after the training interventions, almost the same as pre-training [36]. As far as GSSG is concerned, the same conflicting results were reported between the deep vastus lateralis and the soleus; however, the soleus exhibited a 35–90% higher GSH to GSSG ratio compared to the deep vastus lateralis even after the training period [21,28]. In addition, the soleus demonstrated 17% and 38% higher reduced thiol content compared to the oxidative and glycolytic gastrocnemius, respectively [24].

Regarding GR activity, post-training oxidative gastrocnemius levels were 5%, 68%, 130%, 45% and 20% higher compared to the extensor carpi radialis, triceps, splenius, mixed vastus lateralis and longissimus dorsi, respectively [36]. The soleus exhibited 55–85% greater GR activity than the deep vastus lateralis, while the longissimus dorsi had 20% higher levels compared to the mixed vastus lateralis [21,28,36]. Relating to GPx activity after the training interventions, all the included studies in this review concluded that the soleus, when compared with any other muscle, exhibited by far the highest levels. More specifically, the soleus exhibited higher GPx activity compared to the glycolytic gastrocnemius (15–1590%), the oxidative gastrocnemius (112–365%), the mixed gastrocnemius (406–487%), the rectus femoris (1592–1733%) and the deep (101–243%) and superficial vastus lateralis (1956%) [15,17,21,22,24,26,27,28]. The oxidative gastrocnemius showed higher GPx activity compared to the extensor carpi radialis (39%), triceps (78%), mixed vastus lateralis (112%), splenius (118%), longissimus dorsi (30%) and glycolytic gastrocnemius (253–692%) [15,24,26,36]. Post-exercise GPx mRNA levels in the oxidative gastrocnemius were 65% higher compared to the glycolytic gastrocnemius, while they also appeared 27% higher compared to the soleus [24].

### 3.4. Oxidation Products

Regarding oxidation products post-training, lipid peroxidation, as assessed via MDA levels (TBA assay), was higher in oxidative muscles compared to the glycolytic ones. In particular, the soleus exhibited 450–675% higher levels compared to the glycolytic gastrocnemius, comparable levels compared to the oxidative gastrocnemius and 45–300% higher values in comparison with the deep vastus lateralis [21,28]. Of note, within the gastrocnemius muscle, the oxidative gastrocnemius head shows 325–510% higher MDA levels as opposed to the more glycolytic head glycolytic gastrocnemius post-training [17,21,26,28]. It should, however, be clarified that the two studies that reported higher MDA levels in two glycolytic muscles, namely the plantaris and the extensor digitorum longus, compared to the soleus at rest [29,42], did not apply an exercise training protocol. Instead, the first one implemented a nutritional treatment [29] and showed 42% higher levels of MDA in the plantaris compared to the soleus, while the second one [42] used a model of rheumatoid arthritis and reported similar levels of MDA in the extensor digitorum longus and the soleus post-intervention.

### 3.5. Reactive Species Production

Post-training, NADPH oxidase activity (assessed by H_2_O_2_ production) was 100–190% higher in the soleus compared to the glycolytic gastrocnemius and up to 50% higher in the soleus compared to the oxidative gastrocnemius [16,24]. With respect to mRNA levels of NADPH oxidases in these muscles, NOX2 mRNA levels in the glycolytic and oxidative gastrocnemius were 50–720% and 225–690% higher compared to the soleus, respectively [16,24]. Contrary to NOX2, NOX4 mRNA levels were more than 110% and 30–110% higher in the soleus compared to glycolytic and oxidative gastrocnemius, respectively [16,24]. Finally, DUOX1 mRNA levels, such as NOX2, were 40% and 25% higher in the glycolytic and oxidative gastrocnemius compared to the soleus, respectively [16]. None of the three studies that measured mitochondrial H_2_O_2_ production applied an exercise training protocol. One of them used a high-fat diet protocol and reported much lower H_2_O_2_ production in the soleus compared to the extensor digitorum longus and the epitrochlearis [7], while the other one treated mitochondria obtained from the soleus and the tibialis anterior of young rats with glutamate, malate and antimycin A and reported slightly higher H_2_O_2_ production in the soleus compared to tibialis anterior [43].

### 3.6. Brief Synopsis

In general, the findings after training are largely similar to those without training (Table 2). More specifically, it seems that in post-exercise training (i) antioxidant enzymes still exhibit higher activity in slow-twitch (oxidative) muscles compared to fast-twitch (glycolytic) muscles; (ii) similar to pre-training, within a muscle, heads or regions with higher oxidative capacity present higher antioxidant enzyme activity; (iii) interestingly, the deep vastus lateralis and the oxidative gastrocnemius, which belong to Type IIa muscles exhibit remarkably higher activity and mRNA levels of redox enzymes as well as glutathione levels compared to fast-twitch muscles that belong to Type IIb; (iv) data on mRNA levels of redox enzymes are still conflicting between slow- and fast-twitch muscles after training (e.g., Mn-SOD versus CuZn-SOD and NOX2 versus NOX4); (iv) slow-twitch muscles exhibit higher content of glutathione and reduced thiols compared to fast-twitch muscles; (v) slow-twitch muscles as well as heads and regions with higher oxidative capacity exhibit higher levels of lipid peroxidation (as assessed by the questionable TBA assay).

## 4. Discussion

This is the first review article that aimed to investigate if slow- and fast-twitch skeletal muscles exhibit different redox properties prior to and after an exercise training period, as assessed through antioxidant enzymes (activity and mRNA levels), glutathione metabolism, oxidative stress biomarkers and reactive species production. Most of the evidence presented herein indicates that slow-twitch skeletal muscles, as represented almost exclusively in the literature by the soleus muscle of rats, exhibit higher antioxidant enzyme activity compared to fast-twitch muscles both pre- and post-training. Of note, this was also the case between different heads or regions of fast-twitch skeletal muscles with partially different oxidative capacities, such as between oxidative and glycolytic gastrocnemius heads or deep and superficial vastus lateralis. Contrary to enzyme activity, data on the mRNA levels of antioxidant enzymes are conflicting. The difference between the mRNA levels of antioxidant enzymes and their activity could be partially explained by the intermediary steps between a signal (e.g., exercise-induced reactive species production) that triggers their dynamic expression involving the transcriptional regulatory element ‘Antioxidant Response Element’ (e.g., via the Keap1/Nrf2/ARE system), and their function. These steps include a variety of post-transcriptional and post-translational modifications, such as S-glutathionylation, S-nitrosation and ubiquitination. Furthermore, mRNA levels in trained and untrained states do not necessarily differ at resting conditions but only acutely after exercise. In addition, slow-twitch skeletal muscles present higher glutathione and reduced thiol content, as well as higher lipid peroxidation levels compared to fast-twitch muscles. Finally, mitochondrial hydrogen peroxide production was higher in fast-twitch skeletal muscles compared to slow-twitch muscles prior to exercise training, but no relevant data exist for post-training conditions.

Taking into account (i) the small number of available studies, (ii) the wide heterogeneity between them in terms of research methodology and analysis of redox measurements (i.e., assay used and biomarker analyzed), and (iii) the lack of physiological readouts in the original studies (i.e., muscle function or performance), no causal evidence can be established about a potential metabolic or functional impact of the dissimilar redox profile between slow- and fast-twitch muscles [49]. However, some plausible implications could be speculated. For instance, several exercise studies apply protocols on specific muscle groups or assess their function via isokinetic dynamometry. It would be, therefore, informative to know if isolated slow- and fast-twitch muscles regulate or adjust their redox and energetic metabolism [50] differently during or after an acute resistance and strength [51,52], endurance [53,54] or sprint and high-intensity interval [55,56] exercise sessions.

In the same context, the preliminary findings of the present review may be of interest to researchers conducting in vitro and ex vivo studies using muscle fibers. For instance, when exposing muscle fibers to a reactive species (e.g., hydrogen peroxide or 2,2′-dithiodipyridine) or an antioxidant (e.g., dithiothreitol or glutathione) to investigate the biochemical (e.g., calcium release) and functional (e.g., force production) consequences, we believe that it is critical to know if muscle fibers are Type I or Type II [57,58]. Considering, for example, that reduced glutathione concentration is much higher in Type I fibers compared to Type II, while mitochondrial hydrogen peroxide production is higher in fast-twitch muscles (muscles with more Type II fibers), then any treatment that affects glutathione levels (e.g., use of cysteine; [59]) or mitochondrial redox state (e.g., use of mitoQ; [60]) will possibly lead to different outcomes, depending on the muscle fiber type used.

A similar situation could also be true for studies that apply electrical stimulation to isolated muscle fibers [61]. Despite the fact that great advances have been made in identifying the sources of reactive species during and after exercise [47,62], it would be interesting to know if Type I and Type II fibers exhibit the same production pattern in terms of sources, magnitude and time course. This, in turn, could possibly facilitate the unraveling of the role of reactive species in specific muscle responses and adaptations (e.g., fatigue) or add arguments to the long-lasting debate in the literature regarding the uncertain health-promoting potential of antioxidant supplements [63].

## 5. Limitations

Some limitations of the present work should be acknowledged. First, given the exploratory nature of the present review to investigate, at a preliminary stage, the potentially different redox properties of slow- and fast-twitch muscles, no inferential statistics were performed [64]. Some critical reasons as to why we did not proceed with further analyses were the small number of original studies that compared slow- and fast-twitch muscles side-by-side and the large heterogeneity in their methodology in terms of redox biomarkers evaluated, and especially the analytical methods applied, which is always a matter of concern in redox biology literature [65,66,67,68,69]. The small number of studies that compared muscles side-by-side was also the reason why we did not evaluate other redox biomarkers, such as antioxidant enzyme protein content or DNA oxidation biomarkers (e.g., 8-OHdG). Second, although some original studies implemented an experimental intervention, such as nutrition (e.g., high-fat diet), disease (e.g., rheumatoid arthritis) or aging, we limited our synthesis in young and healthy groups with or without the application of an exercise training protocol. Thus, our findings should not be extrapolated to aged or diseased populations. Third, the soleus was exclusively used by all studies as the archetypical slow-twitch muscle as opposed to the wide spectrum of fast-twitch muscles analyzed. Finally, the post-training data are less or even non-existent for some redox biomarkers, such as for protein carbonyls and peroxiredoxins; thus, further work is warranted to draw safer conclusions for the post-training condition.

## 6. Conclusions

Our analysis demonstrated that, beyond the well-known differences in structural, metabolic and functional characteristics, different types of skeletal muscle fibers also have remarkably different redox profiles. Bearing in mind the fundamental role of redox biology processes in human physiology, the reported redox heterogeneity between muscle fiber types–and, as a result, between slow- and fast-twitch muscles–should be taken into account when conducting exercise or muscle physiology studies.

## Figures and Tables

**Table 1:** Characteristics of the included studies and their findings.

Article	Sample Species	Biomarkers	Percentages (% Differences between Muscles)
Anderson and Neufer 2005 [39]	RATS Male Sprague–Dawley	GPx (activity) [Kinetic, rate-based assay kit]	107.07% higher in the soleus vs. the glycolytic gastrocnemius 2.48% higher in the soleus vs. the oxidative gastrocnemius 102.06% higher in the oxidative vs. the glycolytic gastrocnemius
Capel et al., 2004 [43]	RATS 12 Male Wistar	Mitochondrial protein carbonyl content [Radioactivity with a liquid scintillation analyzer]	**Young** 21.95% higher in the tibialis anterior vs. the soleus
		Glutamate/malate supported H_2_O_2_ release [Fluorescence]	**Young** 165.80% higher in the tibialis anterior vs. the soleus
Criswell et al., 1993 [27]	RATS 36 Female Sprague–Dawley	SOD (activity) [Spectrophotometry]	19.07% higher in the gastrocnemius vs. the soleus 57.51% higher in the soleus vs. the rectus femoris
		GPx (activity) [Spectrophotometry]	401.39% higher in the soleus vs. the gastrocnemius 2190.73% higher in the soleus vs. the rectus femoris
Ehara et al., 2021 [23]	RATS Male Zitter (zi/zi) and SD rats (control)	CAT (mRNA levels) [Quantitative real-time PCR]	299.84% higher in the soleus vs. the extensor digitorum longus
		CuZn-SOD (mRNA levels) [Quantitative real-time PCR]	33.33% higher in the soleus vs. the extensor digitorum longus
		Mn-SOD (mRNA levels) [Quantitative real-time PCR]	3.36% higher in the extensor digitorum longus vs. the soleus
		Prx3 (mRNA levels) [Quantitative real-time PCR]	6.98% higher in the extensor digitorum longus vs. the soleus
		Prx5 (mRNA levels) [Quantitative real-time PCR]	2.17% higher in the soleus vs. the extensor digitorum longus
		Prx6 (mRNA levels) [Quantitative real-time PCR]	5.49% higher in the soleus vs. the extensor digitorum longus
		GPx1 (mRNA levels) [Quantitative real-time PCR]	1121.41% higher in the soleus vs. the extensor digitorum longus
Hirabayashi et al., 2021 [29]	RATS 12 Male Wistar	Mn-SOD (mRNA levels) [SOD Assay kit]	**Control** The same in the plantaris and soleus
		MDA (content) [TBARS Assay kit]	**Control** 53.71% higher in the plantaris vs. the soleus **Malnutrition** 42.5% higher in the plantaris vs. the soleus
Hollander et al., 1999 [22]	RATS 16 Female Sprague–Dawley	CAT (activity) [Spectrophotometry]	422.03% higher in the soleus vs. the deep vastus lateralis 628.60% higher in the soleus vs. the superficial vastus lateralis
		SOD (activity) [Spectrophotometry]	116% higher in the soleus vs. the deep vastus lateralis 80% higher in the soleus vs. the plantaris 100% higher in the soleus vs. the superficial vastus lateralis 8% higher in the superficial vs. deep vastus lateralis
		CuZn-SOD (activity) [Spectrophotometry]	148.18% higher in the soleus vs. the deep vastus lateralis 93.92% higher in the soleus vs. the plantaris 124.88% higher in the soleus vs. the superficial vastus lateralis
		CuZn-SOD (mRNA levels) [Western blot]	200.55% higher in the soleus vs. the deep vastus lateralis 40.93% higher in the soleus vs. the plantaris 1.47% higher in the superficial vastus lateralis vs. the soleus
		Mn-SOD (activity) [Spectrophotometry]	16.94% higher in the soleus vs. the deep vastus lateralis 43.75% higher in the soleus vs. the plantaris 27.77% higher in the soleus vs. the superficial vastus lateralis
		Mn-SOD (mRNA levels) [Western blot]	67.45% higher in the soleus vs. the deep vastus lateralis 55.88% higher in the soleus vs. the plantaris 155.66% higher in the superficial vastus lateralis vs. the soleus
		GPx (activity) [Spectrophotometry]	689.60% higher in the soleus vs. the deep vastus lateralis 1765.49% higher in the soleus vs. the superficial vastus lateralis
Jenkins and Tengi 1981 [14]	RATS AND HAMSTERS 10 Male and Female Sprague– Dawley and Syrian	CAT (activity) [Oxygen cathode method]	121.15% higher in the soleus vs. the extensor digitorum longus (95.65% in hamsters) 58.40% higher in the soleus vs. the oxidative gastrocnemius 304.92% higher in the soleus vs. the glycolytic gastrocnemius
Laughlin et al., 1990 [15]	RATS 78 Male Sprague–Dawley	CAT (activity) [Spectrophotometry]	746.15% higher in the soleus vs. the glycolytic gastrocnemius 285.96% higher in the soleus vs. the oxidative gastrocnemius
		SOD (activity) [Spectrophotometry]	71.59% higher in the medial head triceps brachii vs. the glycolytic long head triceps 35.53% higher in the medial head triceps brachii vs. the oxidative long head triceps 31.84% higher in the oxidative vs. the glycolytic gastrocnemius 41.71% higher in the soleus vs. the glycolytic gastrocnemius 7.48% higher in the soleus vs. the oxidative gastrocnemius
		GPx (activity) [Spectrophotometry]	484.61% higher in the medial head triceps brachii vs. the glycolytic long head triceps 92.4% higher in the medial head triceps brachii vs. the oxidative long head triceps 1215.38% higher in the oxidative vs. the glycolytic gastrocnemius 2676.92% higher in the soleus vs. the glycolytic gastrocnemius 111.11% higher in the soleus vs. the oxidative gastrocnemius
Lawler et al., 1993 [26]	RATS 40 Female Fischer-344 from Harlan Sprague–Dawley (NIA colony)	SOD (activity) [Spectrophotometry]	**Young control** 122.32% higher in the oxidative vs. glycolytic gastrocnemius 258.92% higher in the soleus vs. the glycolytic gastrocnemius 61.44% higher in the soleus vs. the oxidative gastrocnemius **Old control** 64.92% higher in the oxidative vs. glycolytic gastrocnemius 195.52% higher in the soleus vs. glycolytic gastrocnemius 79.18% higher in the soleus vs. the oxidative gastrocnemius
		GPx (activity) [Spectrophotometry]	**Young control** 377.61% higher in the oxidative vs. the glycolytic gastrocnemius 1773.13% higher in the soleus vs. the glycolytic gastrocnemius 292.18% higher in the soleus vs. the oxidative gastrocnemius **Old control** 378.91% higher in the oxidative vs. glycolytic gastrocnemius 1707.22% higher in the soleus vs. the glycolytic gastrocnemius 277.35% higher in the soleus vs. the oxidative gastrocnemius
		MDA (content) [Spectrophotometry]	**Young control** 490.32% higher in the oxidative vs. the glycolytic gastrocnemius 572% higher in the soleus vs. the glycolytic gastrocnemius 13.84% higher in the soleus vs. the oxidative gastrocnemius **Old control** 270.24% higher in the oxidative vs. the glycolytic gastrocnemius 538.01% higher in the soleus vs. the glycolytic gastrocnemius 72.32% higher in the soleus vs. the oxidative gastrocnemius
Leeuwenburgh et al., 1994 [21]	RATS Male Fischer 344	CAT (activity) [Spectrophotometry]	**Young control** 159.49% higher in the soleus vs. the deep vastus lateralis **Adult control** 164.62% higher in the soleus vs. the deep vastus lateralis **Old control** 112.12% higher in the soleus vs. the deep vastus lateralis
		SOD (activity) [Spectrophotometry]	**Young control** 4.59% higher in the soleus vs. the deep vastus lateralis **Adult control** 15.06% higher in the soleus vs. the deep vastus lateralis **Old control** 3.29% higher in the deep vastus lateralis vs. the soleus
		GSH (content) [HPLC]	**Young control** 8.24% higher in the soleus vs. the deep vastus lateralis **Adult control** 38.21% higher in the soleus vs. the deep vastus lateralis **Old control** 63.63% higher in the soleus vs. the deep vastus lateralis
		Total GSH (content) [HPLC]	**Young control** The same in the soleus vs. the deep vastus lateralis **Adult control** 27.27% higher in the soleus vs. the deep vastus lateralis **Old control** 55% higher in the soleus vs. the deep vastus lateralis
		GSSG (content) [HPLC]	**Young control** 50% higher in the deep vastus lateralis vs. the soleus **Adult control** 40% higher in the deep vastus lateralis vs. the soleus **Old control** 27.27% higher in the deep vastus lateralis vs. the soleus
		GSH:GSSG [HPLC]	**Young control** 58.33% higher in the soleus vs. the deep vastus lateralis **Adult control** 92.85% higher in the soleus vs. the deep vastus lateralis **Old control** 107.03% higher in the soleus vs. the deep vastus lateralis
		GR (activity) [Spectrophotometry]	**Young control** 113.11% higher in soleus vs. the deep vastus lateralis **Adult control** 116.49% higher in the soleus vs. deep vastus lateralis **Old control** 70% higher in the soleus vs. the deep vastus lateralis
		GPx (activity) [Spectrophotometry]	**Young control** 180% higher in the soleus vs. the deep vastus lateralis **Adult control** 324.56% higher in the soleus vs. the deep vastus lateralis **Old control** 265.64% higher in the soleus vs. the deep vastus lateralis
		MDA (content) [Spectrophotometry]	**Young control** 11.36% higher in the soleus vs. the deep vastus lateralis **Adult control** 110% higher in the soleus vs. the deep vastus lateralis **Old control** 103.27% higher in the soleus vs. the deep vastus lateralis
Leeuwenburgh et al., 1997 [28]	RATS 21 Female Sprague–Dawley	SOD (activity) [Spectrophotometry]	1.33% higher in the soleus vs. the deep vastus lateralis
		CuZn-SOD (activity) [Spectrophotometry]	14.11% higher in the soleus vs. the deep vastus lateralis
		Mn-SOD (activity) [Spectrophotometry]	42.49% higher in the deep vastus lateralis vs. the soleus
		GSH (content) [HPLC]	214.28% higher in the soleus vs. the deep vastus lateralis
		Total GSH (content) [HPLC]	200% higher in the soleus vs. the deep vastus lateralis
		GSSG (content) [HPLC]	100% higher in the soleus vs. the deep vastus lateralis
		GSH:GSSG [HPLC]	51.65% higher in the soleus vs. the deep vastus lateralis
		GR (activity) [Spectrophotometry]. GPx (activity) [Spectrophotometry] MDA (content) [Spectrophotometry]	97.18% higher in the soleus vs. the deep vastus lateralis 311.53% higher in the soleus vs. the deep vastus lateralis 415.09% higher in the soleus vs. the deep vastus lateralis
Loureiro et al., 2016 [16]	RATS Male Wistar	CAT (activity) [Spectrophotometry]	344.71% higher in the soleus vs. the glycolytic gastrocnemius 67.59% higher in the soleus vs. the oxidative gastrocnemius
		CAT (mRNA levels) [qPCR]	2% higher in the glycolytic gastrocnemius vs. the soleus 44% higher in the oxidative gastrocnemius vs. the soleus
		SOD (activity) [Spectrophotometry]	66.51% higher in the oxidative vs. glycolytic gastrocnemius 53.39% higher in the soleus vs. the glycolytic gastrocnemius 8.55% higher in the oxidative gastrocnemius vs. the soleus
		CuZn-SOD (mRNA levels) [qPCR]	138.97% higher in the oxidative vs. glycolytic gastrocnemius 132.01% higher in the soleus vs. the glycolytic gastrocnemius 3% higher in the oxidative gastrocnemius vs. the soleus
		Mn-SOD (mRNA levels) [qPCR]	54.55% higher in the soleus vs. the glycolytic gastrocnemius 108% higher in the oxidative gastrocnemius vs. the soleus
		Reduced thiols (content) [Spectrophotometry]	36.65% higher in the soleus vs. the glycolytic gastrocnemius 19.80% higher in the soleus vs. the oxidative gastrocnemius
		GPx (activity) [Spectrophotometry]	432.86% higher in the oxidative vs. the glycolytic gastrocnemius 709.85% higher in the soleus vs. the glycolytic gastrocnemius 51.98% higher in the soleus vs. the oxidative gastrocnemius
		GPx 1 (mRNA levels) [qPCR]	1312.42% higher in soleus vs. the glycolytic gastrocnemius 32.45% higher in soleus vs. the oxidative gastrocnemius
		GPx 3 (mRNA levels) [qPCR]	131.48% higher in soleus vs. the glycolytic gastrocnemius 36.79% higher in the soleus vs. the oxidative gastrocnemius
		NADPH oxidase (activity) [Amplex red/horseradish peroxidase assay]	138.04% higher in the soleus vs. the glycolytic gastrocnemius 12.36% higher in the soleus vs. the oxidative gastrocnemius
		NOX2 (mRNA levels) [qPCR]	114.59% higher in the soleus vs. the glycolytic gastrocnemius 66.11% higher in the soleus vs. the oxidative gastrocnemius
		NOX4 (mRNA levels) [qPCR]	169.54% higher in the soleus vs. the glycolytic gastrocnemius 60.25% higher in the soleus vs. the oxidative gastrocnemius
		DUOX1 (mRNA levels) [qPCR]	19.04% higher in the soleus vs. the glycolytic gastrocnemius 2.77% higher in the soleus vs. the oxidative gastrocnemius
Oh ishi et al., 1985 [19]	RATS 10 Male Fisher	CAT (activity) [Spectrophotometry]	**Young** 88.86% higher in the soleus vs. the extensor digitorum longus
		CuZn-SOD (activity) [Spectrophotometry]	**Young** 64.77% higher in the soleus vs. the extensor digitorum longus
		Mn-SOD (activity) [Spectrophotometry]	**Young** 20% higher in the soleus vs. the extensor digitorum longus
		GPx (activity) [Spectrophotometry]	**Young** 256.70% higher in the soleus vs. the extensor digitorum longus
Osório Alves et al., 2020 [24]	RATS Male Wistar	CAT (mRNA levels) [qPCR]	116.74% higher in the glycolytic gastrocnemius vs. the soleus 170.64% higher in the oxidative gastrocnemius vs. the soleus
		SOD (activity) [Spectrophotometry]	79.13% higher in the oxidative vs. glycolytic gastrocnemius 425.21% higher in the soleus vs. the glycolytic gastrocnemius 193.20% higher in the soleus vs. the oxidative gastrocnemius
		CuZn-SOD (mRNA levels) [qPCR]	The same in the soleus vs. the glycolytic gastrocnemius 40.98% higher in the oxidative gastrocnemius vs. the soleus
		Mn-SOD (mRNA levels) [qPCR]	182.5% higher in the glycolytic gastrocnemius vs. the soleus 195% higher in the oxidative gastrocnemius vs. the soleus
		Reduced thiols (content) [Spectrophotometry]	133.57% higher in the soleus vs. the glycolytic gastrocnemius 41.55% higher in the soleus vs. the oxidative gastrocnemius
		GPx (activity) [Spectrophotometry]	74.29% higher in the oxidative vs. glycolytic gastrocnemius 60.91% higher in the soleus vs. the glycolytic gastrocnemius 8.31% higher in the oxidative gastrocnemius vs. the soleus
		GPx (mRNA levels) [qPCR]	1.58% higher in the oxidative vs. the glycolytic gastrocnemius 1.61% higher in the glycolytic gastrocnemius vs. the soleus 3.22% higher in the oxidative gastrocnemius vs. the soleus
		NADPH oxidase (activity) [Amplex Red/Horseradish Peroxidase Assay]	95.45% higher in the glycolytic gastrocnemius vs. the soleus 60.58% higher in the soleus vs. the oxidative gastrocnemius
		NOX2 (mRNA levels) [qPCR]	14.07% higher in the glycolytic gastrocnemius vs. the soleus 61.40% higher in the oxidative gastrocnemius vs. the soleus
		NOX4 (mRNA levels) [qPCR]	0.95% higher in the soleus vs. the glycolytic gastrocnemius 39.62% higher in the oxidative gastrocnemius vs. the soleus
Oyenihi et al., 2019 [42]	RATS 20 Female Sprague–Dawley	TBARS (content) [Spectrophotometry]	2385.20% higher in the soleus vs. the gastrocnemius 60.31% higher in the extensor digitorum longus vs. the soleus 68% higher in the soleus vs. the vastus lateralis
		Total ROS (IU) [OxiSelect™ ROS assay kit]	146.98% higher in the soleus vs. the gastrocnemius 33.98% higher in the soleus vs. the extensor digitorum longus 3.53% higher in the soleus vs. the vastus lateralis
Pereira et al., 1994 [17]	RATS Male Wistar albino	CAT (activity) [Spectrophotometry]	500% higher in the soleus vs. the glycolytic gastrocnemius
		CuZn-SOD (activity) [Spectrophotometry]	235.71% higher in the soleus vs. the glycolytic gastrocnemius
		Mn-SOD (activity) [Spectrophotometry]	150% higher in the soleus vs. glycolytic gastrocnemius
		GPx (activity) [Spectrophotometry]	30.43% higher in soleus vs. the glycolytic gastrocnemius
		TBARS (content) [Spectrophotometry]	20% higher in the soleus vs. the glycolytic gastrocnemius
Picard 2012 [6]		H_2_O_2_ release [Amplex Red System]	115.61% higher in the extensor digitorum longus vs. the soleus 130.68% higher in the extensor digitorum longus vs. the adductor longus 89.94% higher in the mixed gastrocnemius vs. the soleus 103.21% higher in the mixed gastrocnemius vs. the adductor longus 6.98% higher in the soleus vs. the adductor longus
Pinho et al., 2017 [7]	RATS Male Wistar	CAT (activity) [Spectrophotometry]	**Standard chow ** 407.09% higher in the soleus vs. the extensor digitorum longus 173.06% higher in the soleus vs. the epitrochlearis
		SOD (activity) [Spectrophotometry]	**Standard chow** 0.6% higher in the soleus vs. the extensor digitorum longus 39.73% higher in the soleus vs. the epitrochlearis
		GSH (content) [Spectrophotometry]	**Standard chow ** 104.10% higher in the soleus vs. the extensor digitorum longus 17.38% higher in the soleus vs. the epitrochlearis
		Thioredoxin Reductase (activity) [Spectrophotometry]	**Standard chow** 157.97% higher in the soleus vs. the extensor digitorum longus 399.76% higher in the soleus vs. the epitrochlearis
		Mitochondrial H_2_O_2_ emission potential [Amplex UltraRed/Horseradish Peroxidase Assay]	**Standard chow** 400.14% higher in the extensor digitorum longus vs. the soleus 184.49% higher in the epitrochlearis vs. the soleus
Plant et al., 2003 [20]	RATS Female Sprague–Dawley	CAT (activity) [Spectrophotometry]	25.84% higher in the soleus vs. the extensor digitorum longus
Powers et al., 1994 [18]	RATS 72 Female Sprague–Dawley	CAT (activity) [Spectrophotometry]	333.02% higher in the soleus vs. the glycolytic gastrocnemius 210.39% higher in the soleus vs. the oxidative gastrocnemius
		SOD (activity) [Spectrophotometry]	52.03% higher in the oxidative vs. glycolytic gastrocnemius 64.60% higher in the soleus vs. the glycolytic gastrocnemius 8.26% higher in the soleus vs. the oxidative gastrocnemius
		GPx (activity) [Spectrophotometry]	397.29% higher in the oxidative vs. glycolytic gastrocnemius 1658.55% higher in the soleus vs. the glycolytic gastrocnemius 253.62% higher in the soleus vs. the oxidative gastrocnemius
Sen et al., 1992 [36]	DOGS 22 Female beagles	Total GSH (content) [Spectrophotometry]	0.64% higher in the extensor carpi radialis vs. the oxidative gastrocnemius 3.97% higher in the extensor carpi radialis vs. the triceps 7.53% higher in the extensor carpi radialis vs. the splenius
		GR (activity) [Spectrophotometry]	13.82% higher in the oxidative gastrocnemius vs. the extensor carpi radialis 53.78% higher in the oxidative gastrocnemius vs. the triceps 114.12% higher in the oxidative gastrocnemius vs. the splenius
		GPx (activity) [Spectrophotometry]	41.62% higher in the oxidative gastrocnemius vs. the extensor carpi radialis 54.91% higher in the oxidative gastrocnemius vs. the triceps 55.13% higher in the oxidative gastrocnemius vs. the splenius
	RATS 44 Male Han Wistar	Total GSH (content) [Spectrophotometry]	13.33% higher in the oxidative gastrocnemius vs. the mixed vastus lateralis 193.1% higher in the oxidative gastrocnemius vs. the longissimus dorsi
		GR (activity) [Spectrophotometry]	64.29% higher in the oxidative gastrocnemius vs. the mixed vastus lateralis 46.41% higher in the oxidative gastrocnemius vs. the longissimus dorsi
		GPx (activity) [Spectrophotometry]	117.68% higher in the oxidative gastrocnemius vs. the mixed vastus lateralis 18.56% higher in the oxidative gastrocnemius vs. the longissimus dorsi

CAT: Catalase; CuZn-SOD: copper/zinc superoxide dismutase; DUOX1: dual oxidase 1; GPx: glutathione peroxidase; GR: glutathione reductase; GSH: glutathione; GSSG: glutathione disulfide; MDA: malondialdehyde; Mn-SOD: manganese superoxide dismutase; NADPH: nicotinamide adenine dinucleotide phosphate; NOX2: NADPH oxidase 2; NOX4: NADPH oxidase 4; Prx: peroxiredoxins; ROS: reactive oxygen species; SOD: superoxide dismutase; TBARS: thiobarbituric acid reactive substances.

**Table 2:** Characteristics of the included exercise studies and their findings.

Article	Species	Intervention	Biomarkers	Differences between Muscles
Criswell et al., 1993 [27]	RATS 36 Female Sprague–Dawley	Treadmill running (5 days/wk, 12 weeks, 5 min warm-up and cool-down, 20 m/min, 0% grade) Day 1 (15 min, 30 m/min, 0% grade) increased by 5 min/day until ~35 min continuous exercise for the 1st wk Second week (easy and hard days) Continuous group ~70% VO_2 max_ Interval group ~80–95% VO_2 max_	SOD (activity) [Spectrophotometry]	**Continuous** 63.74% higher in the soleus vs. the gastrocnemius 108.95% higher in the soleus vs. the rectus femoris **Interval** 44.50% higher in the soleus vs. the gastrocnemius 98.56% higher in the soleus vs. the rectus femoris
			GPx (activity) [Spectrophotometry]	**Continuous** 405.52% higher in the soleus vs. the gastrocnemius 1591.99% higher in the soleus vs. the rectus femoris **Interval** 487.11% higher in the soleus vs. the gastrocnemius 1733.33% higher in the soleus vs. the rectus femoris
Hollander et al., 1999 [22]	RATS 16 Female Sprague–Dawley	Two-week treadmill exercise before initiation of the training protocol First wk (15 m/min, 0% grade, for 10 min/day, 5 days/week) End 1st wk (16.5 m/min, 0% grade, 30 min) End 5th–10th wk (27 m/min, 12% grade, 2 h/day)	CAT (activity) [Spectrophotometry]	99.39% higher in the soleus vs. the deep vastus lateralis 335.84% higher in the soleus vs. the superficial vastus lateralis
			SOD (activity) [Spectrophotometry]	50% higher in the soleus vs. the deep vastus lateralis 159.09% higher in the soleus vs. the plantaris 128% higher in the soleus vs. the superficial vastus lateralis
			CuZn-SOD (activity) [Spectrophotometry]	75.18% higher in the soleus vs. the deep vastus lateralis 185.71% higher in the soleus vs. the plantaris 123.25% higher in the soleus vs. the superficial vastus lateralis
			CuZn-SOD (mRNA levels) [Western blot]	216.54% higher in the soleus vs. the deep vastus lateralis 143.09% higher in the soleus vs. the plantaris 40.57% higher in the soleus vs. the superficial vastus lateralis
			Mn-SOD (activity) [Spectrophotometry]	13.82% higher in the deep vastus lateralis vs. the soleus 74.07% higher in the soleus vs. the superficial vastus lateralis
			Mn-SOD (mRNA levels) [Western blot]	34.57% higher in the soleus vs. the deep vastus lateralis 89.93% higher in the soleus vs. the plantaris
			GPx (activity) [Spectrophotometry]	183.49% higher in the soleus vs. the deep vastus lateralis 1956.33% higher in the soleus vs. the superficial vastus lateralis
Laughlin et al., 1990 [15]	RATS 78 Male Sprague–Dawley	Modified Stanhope rodent treadmill First 2–6 wks (32 m/min, up an 8% incline, 2 h/day) Last 6 wks (32 m/min, up an 8% incline, 2 h/day)	CAT (activity) [Spectrophotometry]	100% higher in the oxidative vs. glycolytic long head triceps brachii 333.33% higher in the medial head triceps brachii vs. the glycolytic long head triceps 116.66% higher in the medial head triceps brachii vs. the oxidative long head triceps 155% higher in the oxidative vs. glycolytic gastrocnemius 530% higher in the soleus vs. the glycolytic gastrocnemius 147.05% higher in the soleus vs. the oxidative gastrocnemius
			SOD (activity) [Spectrophotometry]	196.89% higher in the medial head triceps brachii vs. the glycolytic long head triceps 89.16% higher in the medial head triceps brachii vs. the oxidative long head triceps 72.95% higher in the oxidative vs. the glycolytic gastrocnemius 81.36% higher in the soleus vs. the glycolytic gastrocnemius 4.86% higher in the soleus vs. the oxidative gastrocnemius
			GPx (activity) [Spectrophotometry]	691.66% higher in the oxidative vs. glycolytic gastrocnemius 1575% higher in the soleus vs. the glycolytic gastrocnemius 111.57% higher in the soleus vs. the oxidative gastrocnemius
Lawler et al., 1993 [26]	RATS 40 Female Fischer-344 from Harlan Sprague–Dawley (NIA colony)	Treadmill running (Speed and grade ~75% VO_2max_) Acute exercise protocol Old group (40 min uphill treadmill running, ~14.5 m/min, 10% grade) Young group (40 min uphill treadmill running, ~22.0 m/min, 10% grade)	SOD (activity) [Spectrophotometry]	**Young trained** 114.28% higher in the oxidative vs. glycolytic gastrocnemius 267.46% higher in the soleus vs. the glycolytic gastrocnemius 71.48% higher in the soleus vs. the oxidative gastrocnemius **Old trained** 42.25% higher in the oxidative vs. glycolytic gastrocnemius 191.83% higher in the soleus vs. the glycolytic gastrocnemius 99.53% higher in the soleus vs. the oxidative gastrocnemius
			GPx (activity) [Spectrophotometry]	**Young trained** 418.98% higher in the oxidative vs. glycolytic gastrocnemius 1589.87% higher in the soleus vs. the glycolytic gastrocnemius 225.60% higher in the soleus vs. the oxidative gastrocnemius **Old trained** 252.61% higher in the oxidative vs. glycolytic gastrocnemius 1539.11% higher in the soleus vs. the glycolytic gastrocnemius 364.84% higher in the soleus vs. the oxidative gastrocnemius
			MDA (content) [Spectrophotometry]	**Young trained** 509.09% higher in the oxidative vs. glycolytic gastrocnemius 448.18% higher in the soleus vs. the glycolytic gastrocnemius 11.11% higher in the oxidative gastrocnemius vs. the soleus **Old trained** 326.54% higher in the oxidative vs. glycolytic gastrocnemius 672.56% higher in the soleus vs. the glycolytic gastrocnemius 81.12% higher in the soleus vs. the oxidative gastrocnemius
Leeuwenburgh et al., 1994 [21]	RATS Male Fischer 344	Quinton small-animal treadmill running—10 weeks Beginning (10 m/min, 0% grade, 10 min/day, 5 days/week) Young group— duration and intensity gradually increased throughout the first 4–6 weeks up to 27 m/min, 15% grade, 60 min/day, 5 days/week ~75% VO_2 max_ Adult group— speed and grade increased slowly in the first 6 wks up to 20 m/min, 10% grade Old group– speed and grade increased slowly in the first 8 wks up to 15 m/min, 5% grade	CAT (activity) [Spectrophotometry]	**Young trained** 115.89% higher in the soleus vs. the deep vastus lateralis **Adult trained** 99.60% higher in the soleus vs. the deep vastus lateralis **Old trained** 108.64% higher in the soleus vs. the deep vastus lateralis
			SOD (activity) [Spectrophotometry]	**Young trained** 38.12% higher in the deep vastus lateralis vs. the soleus **Adult trained** 31.66% higher in the deep vastus lateralis vs. the soleus **Old trained** 18.29% higher in the soleus vs. the deep vastus lateralis
			GSH (content) [HPLC]	**Young trained** 29.25% higher in the deep vastus lateralis vs. the soleus **Adult trained** 61.63% higher in the soleus vs. the deep vastus lateralis **Old trained** 38.46% higher in the soleus vs. the deep vastus lateralis
			Total GSH (content) [HPLC]	**Young trained** 35.29% higher in the deep vastus lateralis vs. the soleus **Adult trained** 47.36% higher in the soleus vs. the deep vastus lateralis **Old trained** 26.08% higher in the soleus vs. the deep vastus lateralis
			GSSG (content) [HPLC]	**Young trained** 70% higher in the deep vastus lateralis vs. the soleus **Adult trained** 16.66% higher in the deep vastus lateralis vs. the soleus **Old trained** 45.45% higher in the deep vastus lateralis vs. the soleus
			GSH:GSSG [HPLC]	**Young trained** 34.18% higher in the soleus vs. the deep vastus lateralis **Adult trained** 73.22% higher in the soleus vs. the deep vastus lateralis **Old trained** 89.84% higher in the soleus vs. the deep vastus lateralis
			GR (activity) [Spectrophotometry]	**Young trained** 84.61% higher in the soleus vs. the deep vastus lateralis **Adult trained** 75.82% higher in the soleus vs. the deep vastus lateralis **Old trained** 73.91% higher in the soleus vs. the deep vastus lateralis
			GPx (activity) [Spectrophotometry]	**Young trained** 101.25% higher in the soleus vs. the deep vastus lateralis **Adult trained** 243.07% higher in the soleus vs. the deep vastus lateralis **Old trained** 228.17% higher in the soleus vs. the deep vastus lateralis
			MDA (content) [Spectrophotometry]	**Young trained** 42.85% higher in the soleus vs. the deep vastus lateralis **Adult trained** 57.69% higher in the soleus vs. the deep vastus lateralis **Old trained** 50.98% higher in the soleus vs. the deep vastus lateralis
Leeuwenburgh et al., 1997 [28]	RATS 21 Female Sprague–Dawley	Quinton rodent treadmill running First week (15 m/min, 0% grade, 10 mm/day, 5 days/wk) End 1st week (16.5 m/min, 0% grade, 30 min) End 4th–10th wk (25 m/min, 10% grade, 2 h/day)	SOD (activity) [Spectrophotometry]	24.52% higher in the deep vastus lateralis vs. the soleus
			CuZn-SOD (activity) [Spectrophotometry]	24.54% higher in the deep vastus lateralis vs. the soleus
			Mn-SOD (activity) [Spectrophotometry]	23.19% higher in the deep vastus lateralis vs. the soleus
			GSH (content) [HPLC]	104.30% higher in the soleus vs. the deep vastus lateralis
			GSSG (content) [HPLC]	50% higher in the soleus vs. the deep vastus lateralis
			GSH:GSSG [HPLC]	40.25% higher in the soleus vs. the deep vastus lateralis
			GR (activity) [Spectrophotometry]	55.84% higher in the soleus vs. the deep vastus lateralis
			GPx (activity) [Spectrophotometry]	180.95% higher in the soleus vs. the deep vastus lateralis
			MDA (content) [Spectrophotometry]	301.53% higher in the soleus vs. the deep vastus lateralis
Loureiro et al., 2016 [16]	RATS Male Wistar	~60% of Maximum Speed Testing (maximal lactate steady state) First week (30 min/day, 5 days/wk) Second week (1 h/day, 5 days/wk) Third week (2 h/day, 5 days/wk)	NADPH oxidase (activity) [Amplex red/horseradish peroxidase assay]	190.73% higher in the soleus vs. the glycolytic gastrocnemius 7.48% higher in the soleus vs. the oxidative gastrocnemius
			NOX2 (mRNA levels) [qPCR]	50.96% higher in the glycolytic gastrocnemius vs. the soleus 225% higher in the oxidative gastrocnemius vs. the soleus
			NOX4 (mRNA levels) [qPCR]	113.76% higher in the soleus vs. the glycolytic gastrocnemius 108.03% higher in the soleus vs. the oxidative gastrocnemius
			DUOX1 (mRNA levels) [qPCR]	41.92% higher in the glycolytic gastrocnemius vs. the soleus 24.59% higher in the oxidative gastrocnemius vs. the soleus
Osório Alves et al., 2020 [24]	RATS Male Wistar	Rodent treadmill Forty-eight hours after the 2 wk acclimation period, one single strenuous exercise session (3 min running stages with constant load at 0.3 km/h initial velocity, with 0.2 km/h rises between stages, until physical exhaustion)	CAT (activity) [Spectrophotometry]	90.01% higher in the soleus vs. the glycolytic gastrocnemius 32.20% higher in the oxidative gastrocnemius vs. the soleus
			CAT (mRNA levels) [qPCR]	124.32% higher in the oxidative vs. glycolytic gastrocnemius 6.73% higher in the glycolytic gastrocnemius vs. the soleus 139.42% higher in the oxidative gastrocnemius vs. the soleus
			SOD (activity) [Spectrophotometry]	291.23% higher in the oxidative vs. the glycolytic gastrocnemius 291.23% higher in the soleus vs. the glycolytic gastrocnemius The same in the oxidative gastrocnemius vs. the soleus
			CuZn-SOD (mRNA levels) [qPCR]	1717.56% higher in the oxidative vs. glycolytic gastrocnemius 812.16% higher in the soleus vs. the glycolytic gastrocnemius 99.25% higher in the oxidative gastrocnemius vs. the soleus
			Mn-SOD (mRNA levels) [qPCR]	98.03% higher in the oxidative vs. glycolytic gastrocnemius 37.25% higher in the soleus vs. the glycolytic gastrocnemius 44.28% higher in the oxidative gastrocnemius vs. the soleus
			Reduced thiols (content) [Spectrophotometry]	38.03% higher in the soleus vs. the glycolytic gastrocnemius 16.60% higher in the soleus vs. the oxidative gastrocnemius
			GPx (activity) [Spectrophotometry]	524.48% higher in the oxidative vs. glycolytic gastrocnemius 251.42% higher in the soleus vs. the glycolytic gastrocnemius 77.70% higher in the oxidative gastrocnemius vs. the soleus
			GPx 1 (mRNA levels) [qPCR]	66.43% higher in the oxidative vs. the glycolytic gastrocnemius 26.59% higher in the oxidative gastrocnemius vs. the soleus
			NADPH oxidase (activity) [Amplex red/horseradish peroxidase assay]	100.71% higher in the soleus vs. the glycolytic gastrocnemius 51.14% higher in the soleus vs. the oxidative gastrocnemius
			NOX2 (mRNA levels) [qPCR]	720.38% higher in the glycolytic gastrocnemius vs. the soleus 691.26% higher in the oxidative gastrocnemius vs. the soleus
			NOX4 (mRNA levels) [qPCR]	114.40% higher in the soleus vs. the glycolytic gastrocnemius 33.86% higher in the soleus vs. the oxidative gastrocnemius
Pereira et al., 1994 [17]	RATS Male Wistar albino	Swimming Sixty minutes daily, 30 °C, 5 days/wk, 8 wks, with extra weight (5% of the body wt) fixed on the tail	CAT (activity) [Spectrophotometry]	150% higher in the soleus vs. the glycolytic gastrocnemius
			CuZn-SOD (activity) [Spectrophotometry]	400% higher in the soleus vs. the glycolytic gastrocnemius
			Mn-SOD (activity) [Spectrophotometry]	541.66% higher in the soleus vs. the glycolytic gastrocnemius
			GPx (activity) [Spectrophotometry]	15.38% higher in the soleus vs. the glycolytic gastrocnemius
			TBARS (content) [Spectrophotometry]	17.85% higher in the soleus vs. the glycolytic gastrocnemius
Plant et al., 2003 [20]	RATS Female Sprague–Dawley	Ten-lane enclosed treadmill Five days/wk, 12 wks 25 min warm-up (9–24 m/min) 60 min running (27 m/min ~65–70% VO_2 max_ 5 min cool-down (12 m/min)	CAT (activity) [Spectrophotometry]	171.55% higher in the soleus vs. the extensor digitorum longus
Sen et al., 1992 [36]	DOGS 22 Female beagles	Ten-track treadmill for dogs First 10 wks (0.5–4.0 km/h, 15% uphill grade) Next 30 wks (5 days/week, 40 km/day, 5.5–6.8 km/h, 15% uphill grade)	Total GSH (content) [Spectrophotometry]	37.88% higher in the oxidative gastrocnemius vs. the extensor carpi radialis 41.4% higher in the oxidative gastrocnemius vs. the triceps 54.16% higher in the oxidative gastrocnemius vs. the splenius
			GR (activity) [Spectrophotometry]	6.16% higher in the oxidative gastrocnemius vs. the extensor carpi radialis 67.1% higher in the oxidative gastrocnemius vs. the triceps 128.31% higher in the oxidative gastrocnemius vs. the splenius
			GPx (activity) [Spectrophotometry]	39.08% higher in the oxidative gastrocnemius vs. the extensor carpi radialis 77.93% higher in the oxidative gastrocnemius vs. the triceps 117.7% higher in the oxidative gastrocnemius vs. the splenius
	RATS 44 Male Han Wistar	Treadmill (for small animals) First 2 wks accustomed to running Third to eighth wk (2.1 km/h, 2 h/day, 5 days/week)	Total GSH (content) [Spectrophotometry]	17.8% higher in the oxidative gastrocnemius vs. the mixed vastus lateralis 207.14% higher in the oxidative gastrocnemius vs. the longissimus dorsi 160.71% higher in the mixed vastus lateralis vs. the longissimus dorsi
			GR (activity) [Spectrophotometry]	44.86% higher in the oxidative gastrocnemius vs. the mixed vastus lateralis 18.52% higher in the oxidative gastrocnemius vs. the longissimus dorsi 22.22% higher in the longissimus dorsi vs. the mixed vastus lateralis
			GPx (activity) [Spectrophotometry]	112.47% higher in the oxidative gastrocnemius vs. the mixed vastus lateralis 29.74% higher in the oxidative gastrocnemius vs. the longissimus dorsi

CAT: catalase; CuZn-SOD: copper/zinc superoxide dismutase; DUOX1: dual oxidase 1; GPx: glutathionem peroxidase; GR: glutathione reductase; GSH: glutathione; GSSG: glutathione disulfide; h:hour; m/min:meters/minute; MDA: malondialdehyde; Mn-SOD: manganese superoxide dismutase; NADPH: nicotinamide adenine dinucleotide phosphate; NOX2: NADPH oxidase 2; NOX4: NADPH oxidase 4; Prx: peroxiredoxins; ROS: reactive oxygen species; SOD: superoxide dismutase; TBARS: thiobarbituric acid reactive substances; wk: week.

## Data Availability

No new data were created or analyzed in this study. Data sharing is not applicable to this article.
